# COMMENTARY ON “EXAMINING AND COMPARING THE CLINICAL CHARACTERISTICS OF ADULTS WITH PERSISTING POST-CONCUSSION SYMPTOMS PRESENTING FOR OUTPATIENT REHABILITATION FOLLOWING A MILD TRAUMATIC BRAIN INJURY OR A MINIMAL HEAD INJURY”

**DOI:** 10.2340/jrm.v58.44545

**Published:** 2026-02-06

**Authors:** Wenjing ZHANG, Linyan ZHAO

**Affiliations:** From the Department of Critical Care Medicine, The Second Hospital of Dalian Medical University, Dalian, Liaoning, China

*To the Editor,*

I would like to extend my sincere congratulations to the authors for their insightful study titled “Examining and Comparing the Clinical Characteristics of Adults with Persisting Post-Concussion Symptoms Presenting for Outpatient Rehabilitation Following a Mild Traumatic Brain Injury or a Minimal Head Injury” published in your esteemed journal.

This well-conducted study presents valuable data on patients with persistent post-concussion symptoms (PPCS), shedding light on an often underrepresented patient group and their rehabilitation needs.

The study comprehensively explores the clinical characteristics and functional limitations of patients referred for outpatient rehabilitation due to PPCS following mild traumatic brain injury (MTBI) and minimal head injury (MHI). By comparing these 2 patient groups in terms of symptom burden and functional outcomes, the authors provide significant insights into the complexities of rehabilitation after mild head injuries (1), adding to the growing body of literature in this area.

While the findings of this study are compelling, I would like to offer several points for consideration that may further enhance the clarity and applicability of the research:

## 1. *Selection bias and generalizability*

The study primarily focuses on patients who have sought rehabilitation services, which may introduce selection bias. As the sample includes only those who have actively sought specialized care, the findings may not fully represent the broader population of individuals with mild head injuries who do not pursue rehabilitation (2). I would encourage the authors to discuss this limitation in greater detail, especially in terms of its potential impact on the generalizability of the results.

## 2. *Further analysis of subgroups and demographic variability*

While the study includes a diverse patient population, additional stratification based on factors such as age, gender, and pre-existing neurological conditions would provide a more nuanced understanding of recovery trajectories. For instance, the study mentions the high proportion of female patients in the sample; a more in-depth exploration of gender differences in symptoms and outcomes could offer valuable clinical insights (3). Stratifying by other variables could further guide the development of more tailored rehabilitation strategies.

## 3. *Methodological considerations – recall bias and early intervention*

The reliance on self-reported pre-injury health conditions, including prior head injuries and mental health status, may introduce recall bias. A more robust method, such as cross-referencing medical records or further investigating the impact of these factors on recovery, would strengthen the study’s findings. Additionally, the role of early intervention in the rehabilitation process is not addressed in detail, but this could be a significant factor in influencing long-term recovery. Future studies may benefit from exploring how early rehabilitation impacts the course of recovery.

## 4. P*sychosocial factors and rehabilitation approaches*

The authors highlight the importance of a biopsychosocial model in rehabilitation for PPCS. However, the role of psychological factors, such as depression, anxiety, and other emotional disorders, warrants further exploration (4). Integrating psychological interventions with physical rehabilitation could improve overall recovery outcomes. Future studies could consider investigating integrated care models that incorporate both physical and mental health components to enhance treatment efficacy.

Building on the foundation laid by this study, I suggest future research focus on the longitudinal outcomes of rehabilitation for PPCS patients, examining whether symptoms resolve over time and identifying factors that predict long-term recovery. Furthermore, evaluating the effectiveness of personalized rehabilitation strategies that address both physical and psychological factors would offer deeper insights into optimizing recovery for these patients.

In conclusion, the authors have made an important contribution to the literature on PPCS and rehabilitation following mild head injuries. Their findings are highly relevant to clinical practice, providing valuable insights that can guide treatment strategies for patients experiencing persistent post-concussion symptoms. I hope that the authors will continue to explore this important area of research, and I look forward to the future publication of their work.

Thank you for considering these comments, and I eagerly await the publication of this important study.
