# Supplementary material for: COMMENTARY ON “EXAMINING AND COMPARING THE CLINICAL CHARACTERISTICS OF ADULTS WITH PERSISTING POST-CONCUSSION SYMPTOMS PRESENTING FOR OUTPATIENT REHABILITATION FOLLOWING A MILD TRAUMATIC BRAIN INJURY OR A MINIMAL HEAD INJURY”
Source: J Rehabil Med. 2026 Feb 6;58:44545. doi: 10.2340/jrm.v58.44545 (PMC12887751; doi:10.2340/jrm.v58.44545)
Supplement: Related texts

# Other: REPLY TO THE COMMENTARY ON “EXAMINING AND COMPARING THE CLINICAL CHARACTERISTICS OF ADULTS WITH PERSISTING POST-CONCUSSION SYMPTOMS PRESENTING FOR OUTPATIENT REHABILITATION FOLLOWING A MILD TRAUMATIC BRAIN INJURY OR A MINIMAL HEAD INJURY”

**Authors:** Linda FORDAL, Grant L. IVERSON, Julia E. MAIETTA, Alexander OLSEN, Cathrine EINARSEN, Simen B. SAKSVIK, Toril SKANDSEN

**Affiliations:** 1 Department of Neuromedicine and Movement Science, Norwegian University of Science and Technology (NTNU), Trondheim; 2 Clinic of Rehabilitation, St. Olav’s University Hospital, Trondheim, Norway; 3 Department of Physical Medicine and Rehabilitation, Harvard Medical School, Boston, MA; 4 Department of Physical Medicine and Rehabilitation, Spaulding Rehabilitation Hospital and the Schoen Adams Research Institute at Spaulding Rehabilitation, Charlestown, MA; 5 Home Base, A Red Sox Foundation and Massachusetts General Hospital Program, Charlestown, MA; 6 Mass General for Children Sports Concussion Program, Waltham, MA, USA; 7 Department of Psychology, Norwegian University of Science and Technology (NTNU), Trondheim; 8 Department of Research and Development, St. Olav’s University Hospital, Trondheim, Norway

We wish to express our sincere appreciation to Dr Wenjing Zhang for her considered and insightful commentary on our recently published study. Dr Zhang’s interest and constructive observations are greatly appreciated. In the following, we respond to the points raised and provide additional context on specific aspects of our work.

## 1. *Selection bias and generalizability*

We acknowledge that our study sample may not represent the full spectrum of individuals with mild head injuries; however, our focus was intentionally placed on those referred to outpatient rehabilitation – patients we routinely encounter in clinical practice and whose needs warrant particular attention. We also recognize that this approach may overlook individuals with similar needs who are not referred, as referral patterns are shaped by factors such as health system organization. In Norway, most patients are referred by their general practitioner to specialized care, with access provided through a government-funded system at minimal or no personal cost. Consequently, our findings are most applicable to settings with comparable healthcare structures, and we acknowledge the potential for selection bias within this context.

## 2. *Further analysis of subgroups and demographic variability*

We appreciate these insightful and valuable reflections. The current article is based on the ongoing Trondheim PPCS study, which will continue to expand in the coming years, providing excellent opportunities to address such research questions as the sample grows.

## 3. *Methodological considerations – recall bias and early intervention*

We fully acknowledge that these concerns are relevant, though they are inherently difficult to address within a clinical study. Inevitably, such data must depend on retrospective accounts of events and healthcare received prior to patient enrolment, and these details are often incompletely documented. Retrieving notes from medical records across the country would be an extensive and highly resource-intensive undertaking, with unstandardized notes prone to omissions or errors. Many of the self-reported previous head injuries or mental health issues were not evaluated by health professionals, yet excluding this information could also introduce bias. The question of mapping early rehabilitation is indeed of great interest, and we share this view; yet it remains challenging to operationalize in real-world settings, where patients engage in a variety of actions based on advice from diverse sources.

## 4. *Psychosocial factors and rehabilitation approaches*

We share the interest in examining psychological and emotional factors in both research and clinical practice. We also agree on the importance of evaluating personalized rehabilitation strategies that address both physical and psychological needs, though such studies are challenging to design. It is also worth exploring whether early, targeted education on return to activity – aimed at reducing fear after mild head injury – could help prevent some cases of PPCS. These approaches are currently being tested and evaluated in our institution.

Finally, an article describing recovery trajectories in the current sample is already in preparation.

## Conclusion

We sincerely appreciate Dr Zhang’s review and her suggestions for further research, which are consistent with our aims and future priorities. We believe her input will contribute to advancing future studies on PPCS, and we look forward to continuing the academic dialogue in this field.
